# Accuracy of the Kato-Katz method and formalin-ether concentration technique for the diagnosis of *Clonorchis sinensis*, and implication for assessing drug efficacy

**DOI:** 10.1186/1756-3305-6-314

**Published:** 2013-10-29

**Authors:** Men-Bao Qian, Peiling Yap, Yi-Chao Yang, Hai Liang, Zhi-Hua Jiang, Wei Li, Jürg Utzinger, Xiao-Nong Zhou, Jennifer Keiser

**Affiliations:** 1 National Institute of Parasitic Diseases, Chinese Center for Disease Control and Prevention, WHO Collaborative Center for Malaria, Schistosomiasis and Filariasis, Key Laboratory of Parasite and Vector Biology, Ministry of Health, Shanghai, People’s Republic of China; 2 Department of Epidemiology and Public Health, Swiss Tropical and Public Health Institute, Basel, Switzerland; 3 University of Basel, Basel, Switzerland; 4 Guangxi Center for Disease Control and Prevention, Nanning, People’s Republic of China; 5 Binyang Center for Disease Control and Prevention, Binyang, People’s Republic of China; 6 Department of Medical Parasitology and Infection Biology, Swiss Tropical and Public Health Institute, Basel, Switzerland

**Keywords:** *Clonorchis sinensis*, Kato-Katz method, Formalin-ether concentration technique, Diagnosis, Efficacy

## Abstract

**Background:**

Clonorchiasis is a chronic neglected disease caused by a liver fluke, *Clonorchis sinensis*. Chemotherapy is the mainstay of control and treatment efficacy is usually determined by microscopic examination of fecal samples. We assessed the diagnostic accuracy of the Kato-Katz method and the formalin-ether concentration technique (FECT) for *C. sinensis* diagnosis, and studied the effect of diagnostic approach on drug efficacy evaluation.

**Methods:**

Overall, 74 individuals aged ≥18 years with a parasitological confirmed *C. sinensis* infection at baseline were re-examined 3 weeks after treatment. Before and after treatment, two stool samples were obtained from each participant and each sample was subjected to triplicate Kato-Katz thick smears and a single FECT examination.

**Results:**

Thirty-eight individuals were still positive for *C. sinensis* according to our diagnostic ‘gold’ standard (six Kato-Katz thick smears plus two FECT). Two FECT had a significantly lower sensitivity than six Kato-Katz thick smears (44.7% *versus* 92.1%; *p* <0.001). Examination of single Kato-Katz and single FECT considerably overestimated cure rates.

**Conclusions:**

In settings where molecular diagnostic assays are absent, multiple Kato-Katz thick smears should be examined for an accurate diagnosis of *C. sinensis* infection and for assessing drug efficacy against this liver fluke infection.

## Background

Clonorchiasis is a chronic neglected disease caused by consumption of raw or undercooked freshwater fish harboring the infective metacercariae of a liver fluke, *Clonorchis sinensis*. An estimated 15 million people are infected worldwide, but the current hotspots of infection are concentrated in the People’s Republic of China (P.R. China), the Republic of Korea and Vietnam [1-7]. Chronic *C. sinensis* infection is associated with liver and biliary conditions, such as gallstone, cholecystitis, cholangitis and cholangiocarcinoma (CCA) [1,3,6,8-11]. CCA is a carcinoma arising in any part of the biliary tree with poor prognosis, causally linked to an infection with *C. sinensis*[12,13], and it has been estimated that several thousand new CCA cases occur annually due to this liver fluke infection [3,6,14].

Chemotherapy with praziquantel is the main strategy to control morbidity due to clonorchiasis [15-17]. Praziquantel is a safe and efficacious drug, usually resulting in high cure and egg reduction rates [18-20]. However, adverse events, including dizziness, sleepiness, headache and diarrhea, following praziquantel administration have been observed, and they might compromise patient compliance. Furthermore, taking into consideration the large-scale use of praziquantel, particularly in the frame of large-scale chemotherapy targeting schistosomiasis [21], there is concern that parasites might develop resistance against praziquantel. The development of new drugs against clonorchiasis and other chronic neglected diseases is thus of high priority [20].

The diagnosis of helminth infections is largely dependent on fecal examination and the most widely used methods are the Kato-Katz technique [22] and the formalin-ether concentration technique (FECT) [23]. The Kato-Katz technique is commonly employed in field surveys due to the relative ease of operation and the possibility to quantify infection intensity, which allows stratification into different intensity classes based on cut-offs provided by the World Health Organization (WHO) [24]. On the other hand, FECT is usually conducted in specialized laboratories for the concurrent diagnosis of helminth and intestinal protozoan infections [25-27].

The aim of this study was to comparatively assess the accuracy of Kato-Katz and FECT for the diagnosis of *C. sinensis*, and to investigate the effect of the diagnostic technique and sampling effort on estimating drug efficacy. Recently, tribendimidine, a drug registered in P.R. China for treating soil-transmitted helminth infection [28], was tested against *C. sinensis* infection in an exploratory clinical trial [29]. Our study was integrated into this trial, which assessed the efficacy of three different treatment regimens; (i) praziquantel (75 mg/kg divided into three doses); (ii) single-dose tribendimidine (400 mg); and (iii) triple-dose tribendimidine (400 mg daily for three days) [29].

## Methods

### Ethical considerations

Our study was approved by the ethics committees in Basel, Switzerland (EKBB; reference nos. 209/09 and 375/11), the Liverpool School of Tropical Medicine (Liverpool, UK; reference no. 12.02RS), and the National Institute of Parasitic Diseases, Chinese Center for Disease Control and Prevention (Shanghai, P.R. China; reference no. 2012–02). A written informed consent form was obtained from each participant. The trial is registered with Current Controlled Trials (identifier: ISRCTN80829842).

### Study participants and sample collection

The study was carried out in the township of Gantang, Binyang county, Guangxi Zhuang Autonomous Region, P.R. China, between June and July 2012. Seventy-four individuals aged 18 years and above with a parasitological confirmed *C. sinensis* infection were included. Before treatment, each participant provided two fresh morning stool samples within 5 days. Three weeks after treatment (praziquantel 75 mg/kg given in three dosages and tribendimidine single- or triple-dose, 400 mg each), two stool samples were again collected from each participant. Sample size calculation, inclusion/exclusion criteria and randomization procedure have been described elsewhere [29].

### Laboratory procedures

Triplicate Kato-Katz thick smears, using standard 41.7 mg templates, were prepared from each stool sample. The Kato-Katz thick smears were examined under a microscope by experienced technicians and the number of *C. sinensis* eggs counted and recorded. Additionally, approximately 1 g of stool from each sample was kept in 10 ml sodium acetate-acetic acid-formalin (SAF) solution, thoroughly broken up and emulsified for subsequent FECT examination [26,27]. Briefly, the SAF solution was composed of 15 g sodium acetate, 20 ml acetic acid and 40 ml 40% formaldehyde in a total volume of 1,000 ml. For microscopic examination, the fixed sample was first re-suspended and strained through a medical gauze into a conical tube. After centrifugation for 1 min at 500× *g*, the supernatant was decanted. If the final sediment contained a volume of more than 1 ml, the first two steps were repeated and part of the suspension removed. Subsequently, 7 ml 0.85% sodium chloride and 2–3 ml diethyl ether were added to the remaining sediment. The tube was closed with a rubber stopper, shaken vigorously (~30 sec) and centrifuged for 5 min at 500× *g*. From the resulting four layers, the first three layers were removed and the remaining layer examined for *C. sinensis* eggs. The number of *C. sinensis* eggs was counted and recorded. When more than 100 eggs for *C. sinensis* were encountered, the laboratory technician noted ‘100+’.

### Statistical analysis

We used STATA version 10.0 (Stata Corp.; College Station, TX, USA) for statistical analysis. As diagnostic ‘gold’ standard, we defined a combination of six Kato-Katz thick smears plus two FECT (two stool samples each subjected to triplicate Kato-Katz thick smears and single FECT). Prevalence, infection intensity (determined by the geometric mean of eggs per 1 g of stool (GM EPG)) and cure rate (CR) for different treatment arms, as assessed by different diagnostic methods and efforts (i.e. single Kato-Katz, triplicate Kato-Katz and single FECT from first stool sample; six Kato-Katz and two FECT), were compared. CR was determined as the proportion of *C. sinensis* egg-positive individuals at baseline, who became egg-negative 3 weeks after treatment. Sensitivity was calculated and presented with a 95% confidence interval (CI). Difference in sensitivity between three or six Kato-Katz thick smears and a single or duplicate FECT was determined by McNemar test on positive individuals. Finally, the agreement between three or six Kato-Katz thick smears and single or duplicate FECT for detecting *C. sinensis* positive or negative individuals, was assessed using Kappa (κ) statistic with the following cut-offs: κ < 0, no agreement; κ = 0.01-0.2, poor agreement; κ = 0.21-0.4, fair agreement; κ = 0.41-0.6, moderate agreement; κ = 0.61-0.8, substantial agreement; κ = 0.81-1.0, almost perfect agreement [27,30,31].

## Results

### Diagnostic accuracy

At the pre-treatment survey, 74 individuals were diagnosed with *C. sinensis* eggs in their stool regardless of the diagnostic technique used (sensitivity for both methods: 100%). Baseline infection intensities obtained with Kato-Katz through different sampling efforts ranged from 2,229 (first Kato-Katz thick smear) to 3,390 GM EPG (six Kato-Katz thick smears). Semi-quantification of infection intensity determined with FECT examination was possible and the results are shown in Table 1.

**Table 1:** **Accuracy of Kato-Katz method (different sampling efforts) and formalin-ether concentration technique (FECT) for the diagnosis of *Clonorchis sinensis *** **at baseline and 3 weeks after treating 74 individuals with a parasitological confirmed infection**

**Diagnostic approach**	**Infection intensity**	**Positives at baseline**			**Positives at 3 week post-treatment follow-up**				**Sensitivity [% (95% CI)]**
		**No.**	** %**	**Infection intensity [EPG (range)]**^**a**^	**No.**	** % (95% CI)**	**Infection intensity [EPG (range)]**^**a**^		
							**Positive**	**False negative**	
Single Kato-Katz^b^	Light	22	29.7	266 (24–960)	20	27.0 (17.4-38.6)	89 (24–696)	n.a	n.a
	Moderate	35	47.3	2,952 (1,080-8,976)	3	4.1 (0.8-11.4)	2,525 (1,512-4,224)	n.a	n.a
	Heavy	17	23.0	19,555 (10,080-47,376)	0	0	n.a	n.a	n.a
	Overall	74	100.0	2,229 (24–47,376)	23	31.1 (20.8-42.9)	138 (24–4,224)	17.6 (4–72)^c^	60.5 (43.5-75.5)
Triplicate Kato-Katz^b^	Light	17	23.0	295 (80–864)	26	35.1 (24.4-47.1)	60 (8–760)	n.a	n.a
	Moderate	38	51.4	2,889 (1,024-9,992)	3	4.1 (0.8-11.4)	2,482 (1,984-3,136)	n.a	n.a
	Heavy	19	25.7	18,390 (10,312-52,240)	0	0	n.a	n.a	n.a
	Overall	74	100.0	2,750 (80–52,240)	29	39.2 (28.0-51.2)	88 (8–3,136)	16.3 (4–72)^c^	76.3 (59.4-88.0)
Six Kato-Katz^b^	Light	13	17.6	350 (124–788)	31	41.9 (30.5-53.9)	35 (4–704)	n.a	n.a
	Moderate	41	55.4	3,050 (1,032-9,428)	4	5.4 (1.5-13.3)	1,808 (1,208-3,636)	n.a	n.a
	Heavy	20	27.0	18,426 (10,656-31,244)	0	0	n.a	n.a	n.a
	Overall	74	100.0	3390 (124–31,244)	35	47.3 (35.6-59.3)	55 (4–3,636)	n.a	92.1 (77.5-97.9)
Single FECT^d^	Light	51	68.9	11.7 (1–100)	13	17.6 (9.7-28.2)	3.6 (1–21)	n.a	n.a
	Moderate	0	0	n.a	0	0	n.a	n.a	n.a
	Heavy	23	31.1	n.a	0	0	n.a	n.a	n.a
	Overall	74	100.0	n.a	13	17.6 (9.7-28.2)	3.6 (1–21)	0.7 (0.5-1)^e^	34.2 (20.1-51.4)^f,g^
Duplicate FECT^h^	Light	35	47.3	19.2 (2–62)	17	23.0 (14.0-43.2)	1.7 (0.5-14.5)	n.a	n.a
	Moderate	22	29.7	n.a	0	0	n.a	n.a	n.a
	Heavy	17	23.0	n.a	0	0	n.a	n.a	n.a
	Overall	74	100.0	n.a	17	23.0 (14.0-43.2)	1.7 (0.5-14.5)	n.a	44.7 (29.0-61.5)^i,j^
‘Gold’ standard^k^	n.a	74	100.0	n.a	38	51.4 (39.4-63.1)	n.a	n.a	100.0

CI, confidence interval; EPG, eggs per 1 g of stool; n.a., not applicable.

^a^EPG is presented as geometric mean.

^b^Light infection, 1–999 EPG; moderate infection, 1,000-9,999 EPG; heavy infection: ≥10,000 EPG.

^c^EPG determined by six Kato-Katz thick smears.

^d^Light infection: ≤100 EPG; heavy infection: >100 EPG.

^e^EPG determined by duplicate FECT.

^f^Difference in sensitivities between three Kato-Katz and single FECT determined by the McNemar test on positive individuals: *p* <0.001.

^g^κ measure of agreement between three Kato-Katz and single FECT taking into account positive and negative individuals: 0.37.

^h^Light infection: both samples ≤100 EPG; moderate infection: 1 sample ≤100 EPG and 1 sample >100 EPG; heavy infection: both samples >100 EPG.

^i^Difference in sensitivities between six Kato-Katz and two FECT determined by the McNemar test on positive individuals: *p* <0.001.

^j^κ measure of agreement between six Kato-Katz and two FECT taking into account positive and negative individuals: 0.33.

^k^Diagnostic ‘gold’ standard is defined as a combination of six Kato-Katz and duplicate FECT.

Three weeks post-treatment, a total of 38 individuals (51.4%) were diagnosed with *C. sinensis* eggs in their stool, according to our diagnostic ‘gold’ standard. Among them, 35 (47.3%) were found to be positive when analyzing six Kato-Katz thick smears, which was more than double the number compared to duplicate FECT (17 positive; 23.0%; *p* = 0.001). The number of positives detected with the first Kato-Katz, triplicate Kato-Katz or single FECT on the first stool sample were 23 (31.1%), 29 (39.2%) and 13 (17.6%), respectively. Six Kato-Katz thick smears detected *C. sinensis* infections with a significantly higher sensitivity than duplicate FECT (92.1% *versus* 44.7%; *p* <0.001). Similarly, triplicate Kato-Katz was significantly more sensitive than single FECT on the first stool sample (76.3% *versus* 34.2%; *p* <0.001). There was poor agreement between six Kato-Katz thick smears and duplicate FECT (κ = 0.33), as well as triplicate Kato-Katz thick smears and single FECT on the first stool sample (κ = 0.37). Post-treatment infection intensities obtained with Kato-Katz through different sampling efforts ranged from 138 (first Kato-Katz thick smear) to 55 GM EPG (six Kato-Katz thick smears). On the other hand, the 13 and 17 positives detected by single and duplicate FECT had infection intensities of 3.6 and 1.7 GM EPG, respectively.

Patients missed by single and triplicate Kato-Katz thick smears but identified as positive according to six Kato-Katz thick smears had infection intensities (based on six Kato-Katz) of 17.6 and 16.3 GM EPG, respectively. Four patients that were negative according to a single FECT had a GM EPG of 0.7 based on duplicate FECT.

### Effect of diagnostic approach on treatment efficacy

Table 2 shows the treatment efficacy, as determined by the observed CR, in relation to the diagnostic approach taken. According to our diagnostic ‘gold’ standard (six Kato-Katz plus duplicate FECT), overall CRs of 44% (single-dose tribendimidine) to 52% (praziquantel) were achieved. Similar CRs were observed with six Kato-Katz thick smears, whereas a single Kato-Katz or FECT resulted in considerably higher CRs. However, no statistically significant differences in the efficacy between praziquantel, tribendimidine single- and triple-dose were observed against *C. sinensis* infection regardless of the diagnostic approach taken.

**Table 2:** **Estimated cure rates of three different treatment regimens against *Clonorchis sinensis *** **infection, according to different diagnostic approaches**

**Diagnostic approach**	**Observed cure rate [% (95% CI)]**		
	**Praziquantel** **(75 mg/kg divided in 3 doses)**	**Tribendimidine** **(400 mg once daily for 3 days)**	**Tribendimidine** **(400 mg once)**
Single Kato-Katz	72.0 (50.6-87.9)	75.0 (53.3-90.2)	60.0 (38.7-78.9)
Triplicate Kato-Katz	60.0 (38.7-78.9)	66.7 (44.7-84.4)	56.0 (34.9-75.6)
Six Kato-Katz	56.0 (34.9-75.6)	58.3 (36.6-77.9)	44.0 (24.4-65.1)
Single FECT	84.0 (63.9-95.5)	87.5 (67.6-97.3)	76.0 (54.9-90.6)
Duplicate FECT	80.0 (59.3-93.2)	79.2 (57.8-92.9)	72.0 (50.6-87.9)
Diagnostic ‘gold’ standard^a^	52.0 (31.3-72.2)	50.0 (29.1-70.9)	44.0 (24.4-65.1)

CI, confidence interval.

^a^Diagnostic ‘gold’ standard is defined as a combination of six Kato-Katz and duplicate FECT.

## Discussion

The control of clonorchiasis and other food-borne trematodiases has gained attention in recent years due to the growing recognition of the public health importance of these chronic parasitic diseases [3,5,17,32]. Chemotherapy is the intervention of choice for morbidity control against clonorchiasis [17,32], as well as other helminthiases [33].

To evaluate anthelmintic drug efficacy, an accurate diagnosis is important [34]. However, the sensitivity of diagnostic methods varies, and it is particularly challenging to identify low-intensity helminth infection with high accuracy [27]. In the present study, we compared the accuracy of the widely used Kato-Katz method (different sampling efforts) with FECT for the diagnosis of *C. sinensis* infection within the frame of a clinical trial. Of note, while the Kato-Katz method was initially developed for the diagnosis of intestinal schistosomiasis [22], it is now routinely used for a wide variety of helminth infections [35], including *C. sinensis* and other fluke infections [36]. In our study, two stool samples were collected at the 3-week post-treatment follow-up from all participants who had a parasitological confirmed *C. sinensis* infection at the baseline survey and had undergone treatment with praziquantel or tribendimidine. The number of individuals identified with *C. sinensis* eggs in their stool after treatment varied considerably as a result of the diagnostic approach used. For example, while a single FECT was able to diagnose all positive cases at baseline (sensitivity: 100%), at the post-treatment follow-up, when low-intensity infections were common, FECT only diagnosed 13 (single FECT) and 17 (duplicate FECT) positive cases, respectively, whereas a total of 35 *C. sinensis*-positive cases were detected based on six Kato-Katz thick smears. Consequently, the sensitivity of FECT at post-treatment was below 50%, whereas six Kato-Katz showed a high sensitivity (92.1%). In addition, FECT examination only allows recording *C. sinensis* eggs in a semi-quantitative manner. The inferiority of FECT compared to the Kato-Katz method reported here corroborates recent results from other studies on another liver fluke infection. Indeed, Soukhathammavong and colleagues [37] and Lovis and collaborators [38] reported that the Kato-Katz method is considerably more sensitive than FECT in detecting *Opisthorchis viverrini* infection. Prior work focusing on the blood fluke *Schistosoma mansoni* speculated that the multiple washing steps in the FECT could influence its sensitivity [39]. Loss of eggs at washing steps might also explain the low EPGs observed with FECT when compared to the Kato-Katz method. However, the use of dyes to stain eggs examined with FECT would allow differential diagnosis of liver and intestinal flukes, which co-exist in our and other study areas in Southeast Asia [40,41].

Our findings have important ramifications on assessing treatment efficacy. Using the insensitive FECT would result in considerably overestimated treatment efficacy against *C. sinensis*. Indeed, the reported CRs using duplicate FECT were 72.0-80.0%, whereas six Kato-Katz showed considerably lower CRs (44.0-58.3%). Hence, the diagnostic approach should be taken into consideration when reporting and interpreting treatment efficacies.

## Conclusions

The Kato-Katz method is more reliable than FECT for the diagnosis of *C. sinensis* and for evaluating drug efficacy (as determined by CR) against this liver fluke infection, particularly when multiple thick smears are analyzed. Although collection of more than one stool sample causes logistic inconvenience and might lower patient compliance, we recommend that at least two stool samples should be obtained in order to achieve an accurate diagnosis at treatment follow-up. FECT cannot be recommended for the diagnosis of clonorchiasis unless it is combined with other techniques, such as polymerase chain reaction (PCR), a highly sensitive approach, which is gaining importance for helminth diagnosis [42-44], but might not be available in resource-constrained settings.

## Competing interests

The authors declare that they have no competing interests.

## Authors’ contributions

Conceived and designed the experiments: JU, XNZ, JK. Performed the experiments: MBQ, PY, YCY, HL, ZHJ, WL, JK. Analyzed the data: MBQ, PY. Contributed reagents/materials/analysis tools: MBQ, PY, YCY, HL. Wrote the paper: MBQ, PY, JU, JK. All authors read and approved the final version of the manuscript.
